# The experience of agency: an interplay between prediction and postdiction

**DOI:** 10.3389/fpsyg.2013.00127

**Published:** 2013-03-15

**Authors:** Matthis Synofzik, Gottfried Vosgerau, Martin Voss

**Affiliations:** ^1^Department of Neurodegenerative Diseases, Hertie-Institute for Clinical Brain Research, University of TübingenTübingen, Germany; ^2^German Research Center for Neurodegenerative Diseases (DZNE)Tübingen, Germany; ^3^Institut für Philosophie, Heinrich-Heine-UniversitätDüsseldorf, Germany; ^4^Department of Psychiatry and Psychotherapy, Charité University Hospital and St. Hedwig HospitalBerlin, Germany

**Keywords:** agency, schizophrenia, delusions of influence, control, internal model, efference copy, comparator model, optimal cue integration

## Abstract

The experience of agency, i.e., the registration that I am the initiator of my actions, is a basic and constant underpinning of our interaction with the world. Whereas several accounts have underlined predictive processes as the central mechanism (e.g., the comparator model by C. Frith), others emphasized postdictive inferences (e.g., *post-hoc* inference account by D. Wegner). Based on increasing evidence that both predictive and postdictive processes contribute to the experience of agency, we here present a unifying but at the same time parsimonious approach that reconciles these accounts: predictive and postdictive processes are both integrated by the brain according to the principles of optimal cue integration. According to this framework, predictive and postdictive processes each serve as authorship cues that are continuously integrated and weighted depending on their availability and reliability in a given situation. Both sensorimotor and cognitive signals can serve as predictive cues (e.g., internal predictions based on an efferency copy of the motor command or cognitive anticipations based on priming). Similarly, other sensorimotor and cognitive cues can each serve as *post-hoc* cues (e.g., visual feedback of the action or the affective valence of the action outcome). Integration and weighting of these cues might not only differ between contexts and individuals, but also between different subject and disease groups. For example, schizophrenia patients with delusions of influence seem to rely less on (probably imprecise) predictive motor signals of the action and more on *post-hoc* action cues like e.g., visual feedback and, possibly, the affective valence of the action outcome. Thus, the framework of optimal cue integration offers a promising approach that directly stimulates a wide range of experimentally testable hypotheses on agency processing in different subject groups.

## Introduction

The experience of agency, i.e., the registration that I am the initiator of my actions, is a basic and constant underpinning of our interaction with the world: whenever we grasp, type, or walk, we register the resulting sensory consequences as caused by ourselves. In the last two decades, several different accounts have been proposed to explain the neurocognitive underpinnings of this experience. While some accounts put a stronger emphasis on processes *preceding* the execution of one's respective action for installing an experience of agency, others more strongly emphasize processes *succeeding* one's action. According to this emphasis (which is, of course, not to be seen as an absolute dichotomy, but rather as two poles on a continuous spectrum), these accounts can be grouped in *predictive* and *postdictive* accounts.

**Figure 1:**
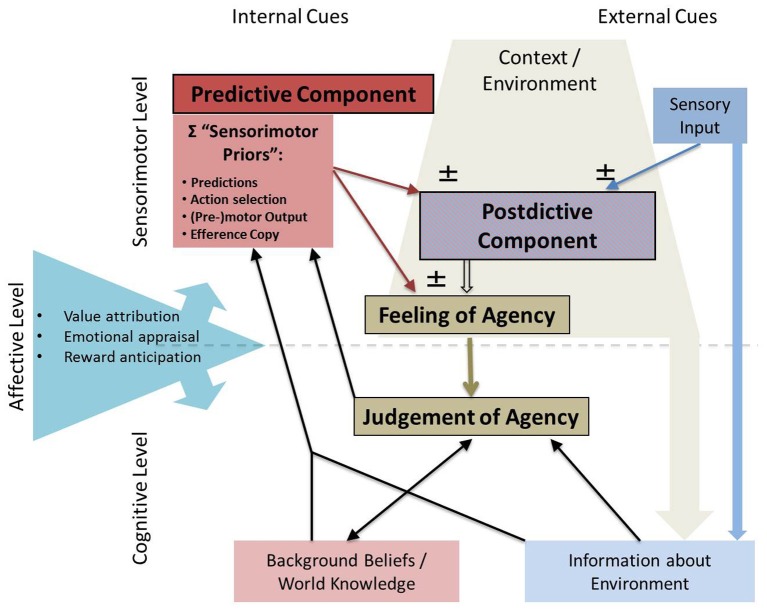
**Proposed account of optimal cue integration underlying the experience of agency.** The sense of agency arises from a complex interplay between a predictive component on the one hand and a postdictive component on the other hand. On a sensorimotor level, the *predictive* component comprises of “sensorimotor priors”: internal cues such as motor predictions (computed in a forward model), action selection, and motor output signals as well as an efference copy of the motor command. Depending on the context and the environment, these internal signals can directly lead to a feeling of agency which only arises due to internal motor command signals. On other occasions, predictions are compared to or integrated with external cues such as sensory input, resulting in a *postdictive* feeling of agency. A low-level, prereflective *feeling of agency* can lead to a more explicit *judgement of agency* on the cognitive level. Here, background information about the environment, internal knowledge about the world or background beliefs have a strong influence on agency judgement. Judgements as well as background beliefs and contextual information in turn can change priors on the sensorimotor level. Furthermore, emotional appraisal, anticipation of reward or punishment or value attribution may influence the weighing of internal or external signals on both the sensorimotor and cognitive level.

Here we discuss the short-comings of either type of account (if seen in isolation) and propose a framework of the experience of agency that will combine both accounts and stimulate manifold experimentally testable hypotheses. This will be illustrated by the example of impaired agency processing in schizophrenia patients suffering from delusions of control. The framework presented here elaborates on and specifies several recent studies that have likewise investigated and proposed mechanisms of an “integration model of agency” (Wegner and Sparrow, 2004; Bayne and Pacherie, 2007; Fletcher and Frith, 2009; Moore et al., 2009a,b; Moore and Fletcher, 2012). However, in contrast to these earlier studies, this framework brings in a new perspective by starting off from an analysis of predictive vs. postdictive accounts, by focussing not only on delusions of control but rather the experience of agency in general [in contrast to e.g., Fletcher and Frith (2009)] and by integrating also very recent results on both predictive processes (e.g., Desantis et al., 2012; Hughes et al., 2013) and *post-hoc* processes. Moreover, it proposes a novel scheme how and on which level different agency cues might be integrated (Figure 1). Finally, we describe the affective valence of an action outcome as a relatively novel self-agency cue, which has not been considered in the original predictive and postdictive accounts and which might explain why delusions of control in schizophrenia patients rarely refer to trivial, non-emotional actions, but rather to very specific actions with high affective and moral value.

## Postdictive vs. predictive accounts of agency

An example for an influential account of postdictive agency processing is Daniel Wegner's famous account (Wegner, 2002, 2003)^1^. Here, the experience of agency is mainly seen as the product of a fallible *post-hoc* inference *during* and *after* the action has occurred, rather than as the result of an infallible direct access to one's cognitive and motor preparation processes *preceding* one's action. According to this notion, the experience of agency for a particular event comes in degrees: it is most strongly, (1) when one's action is the exclusive potential cause of the event (exclusivity), (2) when one has prior thoughts or plans about the action (priority), and (3) when the occurred action matches the action that was planned (consistency). Based on these three criteria, an inference of self-agency is constructed *after* the event has taken place, namely by *postdictive inference*. In this account, low-level motor mechanisms directly related to the motor command and the execution of the action play only a minor role for this inference. Rather, cognitive priors and anticipations, background thoughts, and intention-outcome matching processes (unrelated to very specific and fine-grained characteristics of the actual motor command and the actually executed action) assume a critical role for inferring self-agency. Thus, many inferential accounts—from both Wegner and other authors—also integrate some predictive mechanisms, as they also regard movement priors as important cues for experiencing agency [see e.g., Linser and Goschke (2007)]. However, the experience of agency is nevertheless still essentially seen as the inferential product of a fallible *post-hoc* inference which integrates, inter alia, also cognitive and motor priors. It is not seen as the result of an infallible direct access to one's motor preparation processes *preceding* one's action.

On the other end of the spectrum, accounts elaborating on computational models of sensorimotor integration (Sperry, 1950; von Holst and Mittelstaedt, 1950; von Holst, 1954; Wolpert et al., 1995) hypothesize that the experience of agency for a given action essentially arises from internal motor representations associated with generating the movement that *precede* the action. For example, according to the renowned comparator model (Frith et al., 2000; Blakemore et al., 2002), an internal prediction about the sensory consequences of one's actions is generated on the basis of an efference copy of the motor command. These predicted sensory consequences can be compared with the actual sensory state after that action has been initiated. If the actual sensory state matches the predicted one, it is registered as self-caused. In case of a mismatch, it is registered as externally caused. Although, strictly speaking, this account is also not a purely predictive account of agency—as agency registration here requires the sensory feedback of one's action (and thus also a “postdictive” component) for the comparison process—, the predictive mechanism here plays the critical role. The sensory feedback is only required for comparison purposes and does not *per se* carry the critical information for installing an experience of agency. Thus, in contrast to the inferential accounts of agency, the main emphasis here is not on postdictive inferences but on predictive sensorimotor processes.

## Predictive and postdictive accounts each have major limitations

Within the sense of agency, two levels have to be distinguished: the *feeling of agency*, which consists of a non-conceptual, automatic registration of whether I am the agent or not, and the *judgment of agency*, which is the formation of a belief about who the initiator of the movement was [Synofzik et al., 2008a,b; for a partly different distinction between two levels within the sense of agency see Bayne and Pacherie (2007)]. The automatic registration on the level of feeling can lead to the perception of a particular action or sensory event as self-caused. Subsequently and based on this feeling, a judgment might be established (depending on the demands of the context), which takes into account not only the feeling itself but also context information, background beliefs, general social norms, etc.

Both the predictive and the postdictive accounts have difficulties because they do not respect this distinction. For example, the predictive account based on internal predictions about the sensory consequences of one's movements model might explain the basic, non-conceptual feeling of agency; but it cannot explain the actual conceptual attribution of an action to one's own or somebody else's agency, i.e., the judgement of agency (Synofzik et al., 2008b). This attribution does not depend only on sensorimotor processes, but requires integration of context cues, background beliefs, and *post-hoc* inferences (Synofzik et al., 2008b). In turn, Wegner's postdictive account and many studies supporting this account seem to focus mainly on conscious conceptual *judgements of agency*. These judgements might indeed essentially build on *post-hoc* inferences based on complex cognitive cues such as prior expectations about the task, background beliefs, social interaction, and context estimations. Nevertheless, this postdictive account cannot give an explanation of the feeling-level of agency.

Moreover, Wegner's postdictive account of agency is confronted with several further challenges and biological or explanatory disadvantages:

1. The experience of agency would arise only very *late* in the action process. This would result from the fact that it was necessarily reconstructed only *after* the action (or the event) has occurred. Feedback and cognitive inference mechanisms are known to take long, at least when compared to predictive processes. Such delays would lead to severe failures of sensorimotor systems that need to continuously distinguish whether a sensory event within the ongoing incoming sensory flow is self-caused or not. Even a tiny delay in this process would lead to the perception of the visual environment as instable (Haarmeier et al., 2001; Lindner et al., 2005) or to distracting haptic feedback when interacting with the world (Blakemore et al., 1999).
2. The experience of agency would be a very *fallible* and *error-prone* process. Directly accessible internal motor representations usually present a highly robust and reliable internal action information source. In Wegener's account, however, these motor representations play only a minor role; instead, subjects rather rely on the action context and outcome. Accordingly, the experience of agency would be at constant risk of being misled by *ad-hoc* events and distorting factors in the environment, absent or noisy action feedback, misguided background beliefs, and confusing emotions and evaluations.
3. The information necessary for the experience of agency would not be part of the sensorimotor processing of the action itself. It would be rather *added* to the perception of an action by a *post-hoc* inferential cognitive process.
4. This process seems to function on a *conceptual level*, thus requiring conceptual capacities. However, even relatively simple non-human animals which probably do not have conceptual capacities—like e.g., crickets—are able to distinguish self-produced sensory events from externally produced events (Poulet and Hedwig, 2002, 2006). Thus, this account cannot explain the self/non-self-distinction in these systems, and puts high demands on an explanation of how the experience of agency has phylo- and ontogenetically evolved^2^.

But also the Frith'ian predictive account of agency faces several further challenges and biological or explanatory disadvantages (Synofzik et al., 2008b; Vosgerau and Synofzik, 2012):

1. The output of the comparator model is not only insufficient to explain judgements of agency. In some instances, it can also not fully explain the direct non-conceptual perception of one's actions. A recent study by Wilke and colleagues shows that the perception of one's actions is—in addition to the comparison between internal predictions and sensory feedback—also modulated by external cues presented *post-hoc* (here: the affective valence of action outcomes) (Wilke et al., 2012).
2. A comparator processing might, at least in some instances, not even be *necessary* for the experience of agency. For example, in a “helping hands” pantomime task, subjects experienced high degrees of agency for movements that were performed by another agent, when the other agent's hands appeared in the place where subjects'hands would normally appear and when subjects could hear instructions previewing each movement (Wegner et al., 2004). Since subjects' own arms remained passive, there was most plausibly no efference copy tied to one's motor command that could be used for a specific and detailed prediction about the upcoming event (but, if at all, only a general cognitive anticipatory or intentional state). This finding demonstrates that internal predictions (which are only issued in case of active movements) are not necessary to induce an experience of agency, but external cues (here: externally provided prior instructions) can substitute it. In fact, this particular finding is rather in line with a *postdictive inferential* account of agency.
3. The comparator model account might explain some instances of the experience of agency, but needs various adjustments for many other instances (Carruthers, 2012; Vosgerau and Synofzik, 2012). For example, with respect to priming studies, “the amount of modification to the [comparator] model needed is becoming incredibly large and none of these modifications is predicted by the initial [comparator] model” (Carruthers, 2012, p. 43). Thus, it not only remains questionable whether it is indeed possible to integrate all different adjustments into a coherently adjusted comparator model; the comparator model does also not specify a number of problems, thus making various different adjustments possible and necessary, which cannot be extrapolated from the comparator model itself anymore (Vosgerau and Synofzik, 2012).

## Optimal cue integration: combining predictive and postdictive agency cues

If evaluated in separation, both the predictive and the postdictive account face severe challenges and limitations. And, indeed, there is increasing evidence that the experience of agency does not result from *either* predictive *or* postdictive processes, but that *both* types of processes contribute to the experience of agency, and that they do so in a closely interacting way. For example, Kühn and colleagues suggested that agency judgements incorporate early information processing components (based on the finding that agency judgements were predictable already by the P3a component of tone event-related potentials), and are not purely reconstructive, *post-hoc* evaluations generated only at time of judgement (Kuhn et al., 2011). In turn, as mentioned above, the perception of one's actions is not fully determined by predictive motor processes, but also modulated by external cues presented *post-hoc*, like e.g., the affective valence of the action outcome (Wilke et al., 2012).

But how might the brain integrate predictive and *post-hoc* cues to form a valid and reliable experience of agency for a given sensory event in a particular situation? A proposal of *optimal cue integration* has recently emerged: the brain constantly integrates several different authorship cues and weights each cue according to its relative reliability in a given situation (Synofzik et al., 2009, 2010; Synofzik and Voss, 2010). The reliability of a cue would be low if its variance is high; in turn, its reliability would be high if it is present in a very salient way and/or highly precise. This notion follows the framework of optimal cue integration established in the field of object perception: according to this framework, no single information signal is powerful enough to convey an adequate representation of a certain perceptual entity under all everyday conditions. Instead, depending on the availability and reliability of a certain information cue, different combination and integration strategies should be used to frame the weighting of sensory and motor signals. Usually, predictive efferent signals such as internal predictions serve as the most reliable and robust agency cues, as they usually provide the fastest and least noisy information about one's own actions (Wolpert and Flanagan, 2001). However, in some situations and subjects, other cues might outweigh or even replace these efferent signals to install a basic registration of agency. For example, if predictive cues like internal predictions are weak or imprecise, *post-hoc* cues like the action feedback or the action outcome should receive a higher weight for determining one's experience of agency. In other words: the variance within one agency cue should be directly related to the reliance on another. Thus, optimal cue integration might not only allow robust perception of objects and the world (Ernst and Banks, 2002; Ernst and Bulthoff, 2004) and efficient sensorimotor learning (Kording and Wolpert, 2004), it could also provide the basis for subjects' robust, and at the same time flexible, agency experience in variable contexts (Synofzik et al., 2009; Synofzik and Voss, 2010; Moore and Fletcher, 2012).

Predictive cues entering the cue integration process are in a sensorimotor format and can consist of e.g., an efference copy, internal predictions based on an efferency copy of the motor command (Frith et al., 2000) or sensorimotor predictions based on automatic associations [e.g., through subliminal priming priming (Wegner, 2003; Wegner et al., 2004; Aarts et al., 2005)]. We refer to these different predictive components as “sensorimotor priors” (see Figure 1). Some sensorimotor priors can also be influenced by cognitive cues like background beliefs or knowledge about the world [e.g., motor processing or sensorimotor predictions can by influenced by autosuggestion or through supraliminal priming (Wegner et al., 2004; Aarts et al., 2005) or through prior causal beliefs induced by contextual information (Desantis et al., 2011)] (see Figure 1). Also the postdictive component can contain sensorimotor cues, e.g., the visual feedback of the action (Synofzik et al., 2010) or feedback in other sensory modalities (including proprioception). Both predictive and postdictive components can contribute to the feeling of agency, which operates on a non-conceptual sensorimotor level (see Figure 1).

On the conceptual cognitive level, a judgement of agency is formed. This is largely based on the feeling of agency, but also takes into account cognitive cues like background beliefs and information about the environment [e.g., the *post-hoc* observation that I am the only person in the room (cf. de Vignemont and Fourneret, 2004)]. At both levels—the level of feeling and the level of judgement of agency—the cue integration process can be modulated by affective components (e.g., affective valence of the action outcome [Wilke et al., 2012] (see Figure 1)). The context and the environment have a direct influence on the weighting of postdictive sensorimotor cues (e.g., lighting conditions on the reliability of vision), and a more indirect influence on the formation of the judgment of agency via cognitive representations of the environment (see Figure 1).

If understood in this way, optimal cue integration provides a unified framework to explain many findings from recent studies of agency, such as priming studies. For example, in the abovementioned study by Moore et al. (2009a), which combines intentional binding and priming, passive movements can be seen as an instance where internal predictions are not available for the system. The optimal cue integration approach would now predict that external cues (e.g., primes) should receive a higher weight for determining the experience of agency. This is exactly what the authors observed: primes modulated perceived intervals for both active and passive movements, but the modulation was greatest for passive movements (Moore et al., 2009a; Synofzik et al., 2009).

This finding, however, has to be interpreted with caution as—in contrast to a long-standing assumption—intentional binding (present in the active condition) does not necessarily reflect a signature of agency. As we have argued earlier (Synofzik et al., 2009), the fact that perceived time intervals between movement and effect were decreased by priming also in case of involuntary movements opens up the possibility that the binding between movement and effect might not be specific to agency and intentionality, but can also present—at least in part—a more unspecific effect linked to temporal binding between two events (in this case between the two congruent sounds, i.e., between prime and effect). Indeed, recent studies suggest that intentional binding is neither linked specifically to motor predictive processes (Desantis et al., 2012; Hughes et al., 2013) nor to agency (Buehner and Humphreys, 2009; Buehner, 2012; Dogge et al., 2012), but rather to causality in general. However, even if the phenomenon of binding of movements to their effects was not due to motor predictive processes, it could still contribute to the experience of agency, for instance, by accentuating subject's perception of the temporal contiguity between movements and their effects (Desantis et al., 2012). Since this accentuation would probably be higher for active than for passive movements, it might also serve as a stronger agency cue in active than in passive movements. Correspondingly, the optimal cue integration approach would predict that subjects' experience of agency would be more open to modulation by external primes in the passive condition than in the active condition. This interpretation would still be compatible with the findings by Moore et al. (2009a).

If internal predictions do not allow to predict the effect of an action—e.g., because of a low contingency between action and effect—, the optimal cue approach would predict that other cues (e.g., primes) should be given more weight for the registration of agency. These additional cues, however, should not receive particular weight if internal predictions serve as a sufficiently reliable predictor for an upcoming event.

This hypothesis was investigated by Gentsch et al. (2012). Subjects had to press a key, which was followed by a certain visual outcome on a computer screen (arrows pointing up or down) with high (75%) or low (50%) contingency, and which was preceded by a congruent or incongruent prime. In case of high contingency, subjects could reliably predict the visual outcome (arrow pointing up or down), and they should not need to rely on the prime. In case of low contingency, however, they could not do so; here they should rely also on the prime. This is exactly what the authors observed: in the low contingency condition, but not in the high contingency condition, priming had an effect on the judgement of the causal strength between action and effect. However, this effect was not found on the level of the cortical N1 response to actively generated feedback, which the authors take as a measure for the feeling of agency. Here priming influenced the response independent of the contingency between action and effect. However, the cortical N1 response might not be a measure of the feeling of agency [as suggested by the authors (Gentsch et al., 2012)], but only of one of the cues—in this case a sensorimotor prediction based on priming as opposed to the motor prediction based on implicit learning of contingencies. On this interpretation, the sensorimotor prediction would be weighted high if no motor predictions are present (low-contingency) and low if motor predictions are present (high-contingency).

## Integration of predictive and *post-hoc* cues in schizophrenia patients

Schizophrenia patients suffering from delusions of influence can be seen as “pathophysiology model” for agency processing, i.e., they provide a window to the processes underlying one's self-attribution of actions. In particular, they illustrate how predictive and *post-hoc* cues of agency are both integrated according to the principles of cue integration (Fletcher and Frith, 2009; Synofzik et al., 2010).

Schizophrenia patients with delusions of influence feel that their actions are no longer controlled by themselves. Sometimes they not only experience their actions as not self-caused, leading only to a vague and strange experience, but also attribute them to some specific other agents (e.g., to a friend, neighbor, or the devil) (Frith, 1992). How can this experience be explained by the optimal cue integration approach? Although several studies that argue for a close link between delusions of influence and a deficit in internal motor predictions have to be interpreted with caution^3^, two recent studies using very different paradigms—namely a visual distortion paradigm and an intentional binding paradigm—provide complementary evidence that schizophrenia patients might indeed show imprecise internal predictions about the sensory consequences of their own actions (Synofzik et al., 2010; Voss et al., 2010). Both studies also showed that this deficit correlated with the severity of the psychopathology: the higher the imprecision in predicting the sensory consequences of one's own actions, the higher the score for delusions of influence (Synofzik et al., 2010). Similar results using an intentional binding paradigm were found for patients in a putative psychotic prodromal stage, suggesting a disturbance of agency already early in the course of the disease (Hauser et al., 2011a). Following the optimal cue integration approach, imprecise predictions should prompt the perceptual system to rely more strongly on *post-hoc* cues in order to receive a more reliable account of one's own actions. And indeed, the study by Synofzik and colleagues found that schizophrenia patients relied more on *post-hoc* information about their actions (in the study: vision) (Synofzik et al., 2010). Similarly, another study investigating schizophrenia patients, as well a group of patients with a putative psychotic prodrome, showed that both patient groups, compared to healthy individuals, relied more strongly on external additional sensorimotor cues to agency in an ambiguous situation, where the reproduction of a drum-pad sequence had to be judged with respect to self-agency (Hauser et al., 2011b).

The approach of optimal cue integration might thus provide a common basis for the various misattributions of agency in schizophrenia patients, including their episodic nature (Synofzik and Voss, 2010; Synofzik et al., 2010). In schizophrenic patients with delusions of influence, internal predictions about the sensory consequences of one's own actions could be frequently imprecise and non-reliable. Patients should therefore be prompted in certain situations to rely more on (seemingly more reliable) alternative cues about self-action. These might either be *post-hoc* (e.g., vision, auditory input, affective valence of the action outcome, or postdictive thoughts), or predictive (e.g., prior sensorimotor expectations based on specific background beliefs or prior emotional appraisal of the situation). The stronger weighting of these alternative cues could help patients to avoid misattribution of agency for self-produced sensory events in the case of imprecise internal action-related predictions. However, as a consequence of giving up the usually most robust and reliable internal action information source, i.e., internal predictions, the sense of agency in psychotic patients is at constant risk of being misled by *ad-hoc* events, invading beliefs, and confusing emotions and evaluations. In other words: schizophrenia patients would be at constant risk of becoming “a slave to every environmental influence” (Frith, 1994, p. 151)—and to every affective and moral *ad-hoc* evaluation. Different agency judgement errors may result: patients might over-attribute external events to their own agency whenever these more strongly weighted alternative agency cues are not veridical and misleading, as is the case in delusions of reference (also referred to as “megalomania”). Conversely, if alternative cues are temporarily not attended or unavailable, patients might fail to attribute self-produced sensory events to their own agency and instead assume external causal forces (as is the case in delusions of influence). A context-dependent weighted integration of imprecise internal predictions and alternative agency cues may therefore reflect the basis of agency attribution errors in both directions: over-attribution, as in delusions of reference/megalomania, and under-attribution, as in delusions of influence (Synofzik and Voss, 2010; Synofzik et al., 2010).

Agency attribution in patients with delusions of influence usually has a very specific semantic content, differing from individual to individual (e.g., a delusional attribution of an action to a particular neighbor, relative, or religious entity), and fails only episodically and only in certain contexts. The cue integration approach might also explain these features: (1) an imprecision in efferent action-related information leads generally to a fluctuating, unreliable basis on which the sense of agency is built, prompting schizophrenia patients to rely more on other alternative cues, which might be misleading in some situations. (2) An altered weighting of affective cues and the well-established disturbances in formal thinking^4^ in schizophrenia will then lead to an unbalanced and disturbed integration of different agency cues with a lack of coherency and consistency. (3) This leads to the formation of a delusional belief, resulting from an individual's weighting of cognitive and affective cues in a particular situation and the individual's personal background beliefs and history.

This would also explain why delusions of control do mostly not refer to trivial, non-emotional actions in daily life (e.g., brushing teeth or typing on a computer), but mainly to very specific, singular actions with high affective and/or moral value. Mostly, they refer to actions that are morally and socially not acceptable or at least negatively connoted, e.g., causing an accident, hurting someone, or behaving inappropriate in the presence of one's peers. Here the affective and moral valence gains major influence on both the sensorimotor and the cognitive level (which might lead to modulated predictions and perception as well as to specific negative beliefs), such that the action is consequently not attributed to one's own agency.

## Conclusions

The registration of being the initiator of one's own actions seems to arise from a dynamic interplay between predictive cues and postdictive cues. These can be in a sensorimotor format (e.g., internal predictions about the sensory consequences of one's actions or visual feedback) or in a cognitive format (e.g., background beliefs or information about the environment). The cues are not mutually exclusive, but used in combination according to their respective reliability to establish the most robust agency representation in a given situation. The cues and the weighting itself can be modulated by factors of the environment as well as by affective factors (e.g., emotional appraisal or reward anticipation).

So far, only limited and preliminary experimental evidence is available to support this novel framework of agency awareness (Moore et al., 2009a; Synofzik et al., 2010; Hauser et al., 2011b; Gentsch et al., 2012; Moore and Fletcher, 2012). Yet this framework stimulates a wide range of questions and hypotheses on agency processing in different subject groups that will be experimentally testable:

1. In healthy subjects, which combination and which strength of predictive or postdictive cues is necessary to override internal predictions in installing (or rejecting) a sense of agency?
2. Does optimal cue integration with respect to agency really occur by a relative continuous shifting of weights along a gradual scale, or are there threshold effects?
3. Are *post-hoc* cues (like e.g., visual feedback) similarly weighted like predictive cues (e.g., primes)? Or is there a general bias toward a stronger weighting of one of these types of cues?
4. How do certain background conditions modulate the weighting of each cue? For example, do conditions like e.g., stress, emotional arousal, or social distress lead to a stronger weighting of postdictive cues?
5. Is there a general difference between how cues are integrated on the level of feeling vs. the level of judgement of agency?
6. In schizophrenia patients, do imprecise predictions lead to a similar over-reliance on predictive cues (like e.g., primes) as on *post-hoc* cues (like e.g., visual feedback), or receive postdictive cues generally a stronger weight?
7. Are schizophrenia patients particularly prone to modulations of the weighting by affective factors? Or do they just show a greater reliance on *post-hoc* cues?
8. Do neurological patients with e.g., cerebellar or parietal lesions also show imprecise internal predictions about the sensory consequences of their actions? If yes, can a difference in their cue integration explain why they do not also show delusions of agency (like schizophrenia patients)? For example, are they less prone to over-rely on *post-hoc* cues? Or is it simply the lack of formal thought disorder, which preserves their cue integration process and thus their sense of agency?

### Conflict of interest statement

The authors declare that the research was conducted in the absence of any commercial or financial relationships that could be construed as a potential conflict of interest.

## Footnotes

^1^For the following summary of these accounts, we were inspired by the nice overview and comparison given at http://en.wikipedia.org/wiki/Inferring_self-agency (Accessed 08/11/2012).

^2^The self-external distinction which also occurs in simple animals and during many continuous sensorimotor operations in humans should, of course, not be equated with the experience of agency, but is only a necessary (yet not sufficient) condition for this experience. This distinction might build the basis and trigger an experience of agency, but is, in itself, only a very basic, mostly non-conscious registration of a low-level registration system (Vosgerau and Newen, 2007; Synofzik et al., 2008a).

^3^A deficit of motor predictive mechanisms in schizophrenia is often inferred from studies that observe abnormal sensory attenuation and intentional binding in these patients. However, it has been argued that the contrasts used by these studies appear to differ in a number of processes other than motor prediction, such as temporal prediction and temporal control (Hughes et al., 2013). Also many other studies commonly taken as support for the notion of prediction deficits in schizophrenia patients with delusions of control can, in fact, not directly explain delusions of control (Synofzik et al., 2008a,b, 2010).

^4^Features of formal thought deficits in schizophrenia patients which are probably particularly relevant for the formation of delusional beliefs include deficits in probabilistic reasoning and a premature “jumping to conclusions.” Based on these deficits, patients might not give an adequate probabilistic weight to each agency cue and reach conclusions on the basis of significantly less evidence than healthy subjects and express more confidence in their decisions (Fletcher and Frith, 2009). This might explain the clinical observation that “patients all too easily develop false beliefs, which they then hold with great confidence and immunity to any counter evidence” (Fletcher and Frith, 2009, p. 50).
